# Pericecal hernia in a pediatric patient: Case report and literature review

**DOI:** 10.1016/j.ijscr.2019.06.043

**Published:** 2019-06-26

**Authors:** Loay M. AlJaberi, AlaaEddin K. Salameh, Raed M. Mashalah, Ayman AbuMaria

**Affiliations:** Bethlehem Arab Society for Surgery and Rehabilitation, Bethlehem, Palestine^1^

**Keywords:** Small bowel obstruction, Internal hernia, Pericecal hernia, Pediatric hernia, Case report

## Abstract

**Introduction:**

Pericecal hernia is a rare cause of small bowel obstruction. They tend to affect the adult population more commonly but also may occur in the pediatric age group.

**Case presentation:**

We herein present a 16-year-old male who presented with 4 days of abdominal pain and distention. CT scan revealed evidence of dilated small bowel loops with evidence of ischemia. A laparotomy was performed and revealed a 20 cm segment of distal ileum protruding through the superior ileocecal recess. Ileocecectomy with primary side to side anastomosis was successfully performed and the patient recovered uneventfully after the procedure.

**Discussion:**

Although internal hernias may have a congenital etiology, most of the reported cases have occurred during adulthood and its presentation in childhood is uncommon. CT scan is the key for internal hernia diagnosis and management as it provides information about the presence of bowel obstruction, strangulation, and ischemia.

**Conclusion:**

High index of clinical suspicion and early intervention saves the patient from extensive bowel resection and other serious complications.

## Highlights

• Pericecal hernia is a rare type of internal hernias accounting for 0.1–6.6% of all internal hernias.
• Pericecal hernia is rare in pediatric patients, clinical presentation depends on the degree of bowel obstruction.
• Keeping in mind the radiation risks. CT scan is the key for internal hernia diagnosis and management.
• High index of clinical suspicion and early intervention saves the patient from extensive bowel resection.

## 1 Introduction

Internal hernias are characterized by the protrusion of a viscus or a solid organ, partially or completely through a peritoneal, mesenteric, omental, or a diaphragmatic aperture. They are classified into congenital and acquired hernias. Congenital hernias are usually caused by abnormal anatomic structural defects such as paraduodenal, transmesenteric, pericecal, transmesosigmoidal, supra- or perivesical, through Winslow’s foramen, omental hernia, and rarely, hernia through the broad ligament, the mesoappendix, or the mesentery of a Meckel’s diverticulum [1]. In contrast, acquired hernias happen after surgical interventions or trauma.

The reported incidence of internal hernias ranges from 0.6% to 5.8% of all small bowel obstructions [2]. Internal hernias may pose a diagnostic challenge preoperatively because of their nonspecific presentation. Abdominal computed tomography (CT) gives information if the obstruction is partial or complete, its location, and helps in determining the surgical management. Taking in consideration the high dose of radiation of abdominal CT, and especially in children; it is important for the physician to weigh the risks and benefits of CT for the diagnosis and management of small bowel obstruction before obtaining imaging.

Pericecal hernias are responsible for 0.1–6.6% of internal hernias [3]. In most cases, ileal loops herniate through the defect and occupy the right paracolic gutter. Clinical diagnosis of pericecal hernias is not always clear. Symptoms and physical examination may indicate acute small bowel obstruction, but in chronic incarceration diagnoses may be confused with inflammatory bowel disease, appendiceal disorders, or other causes of small bowel obstruction [4].

This case report has been reported in line with the SCARE criteria [5].

## 2 Case presentation

**Fig. 1:**
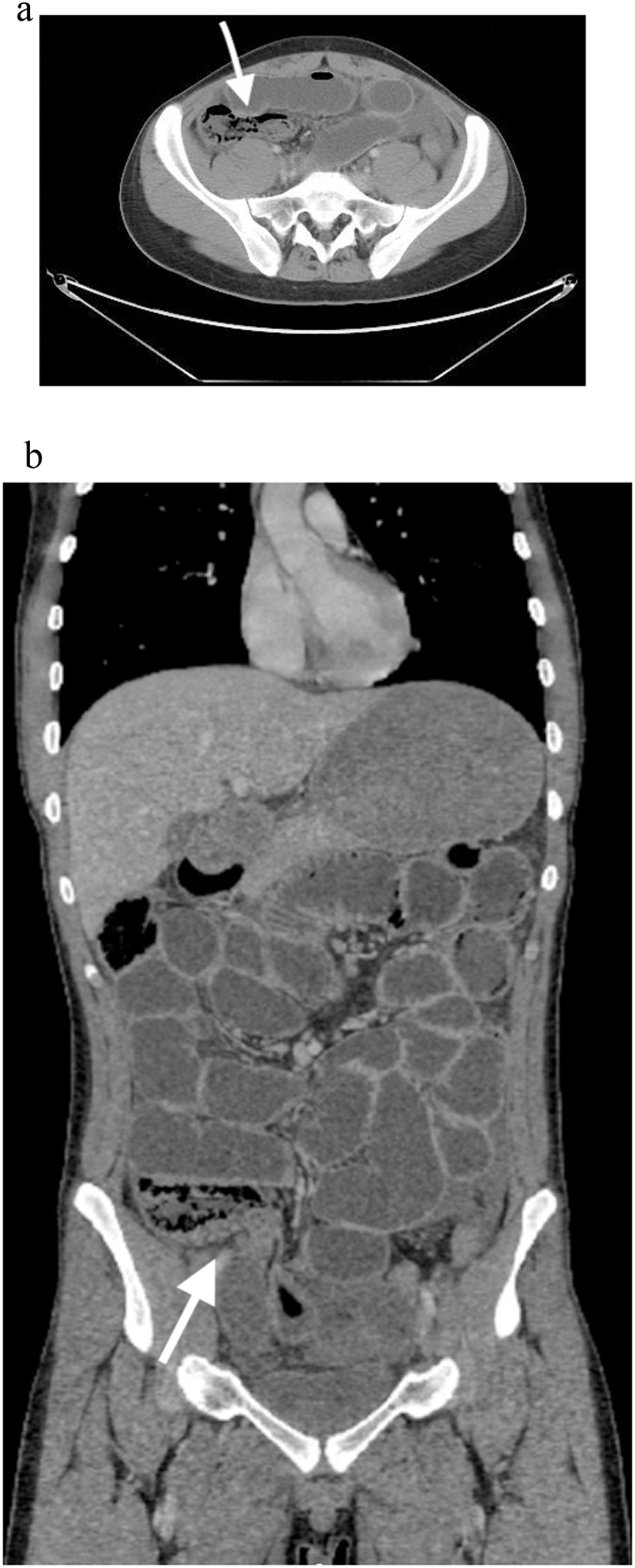
**A and B:** CT scan of the abdomen demonstrating dilated small bowel, transitional zone of the obstruction, and evidence of pneumatosis intestinalis.

**Fig. 2:**
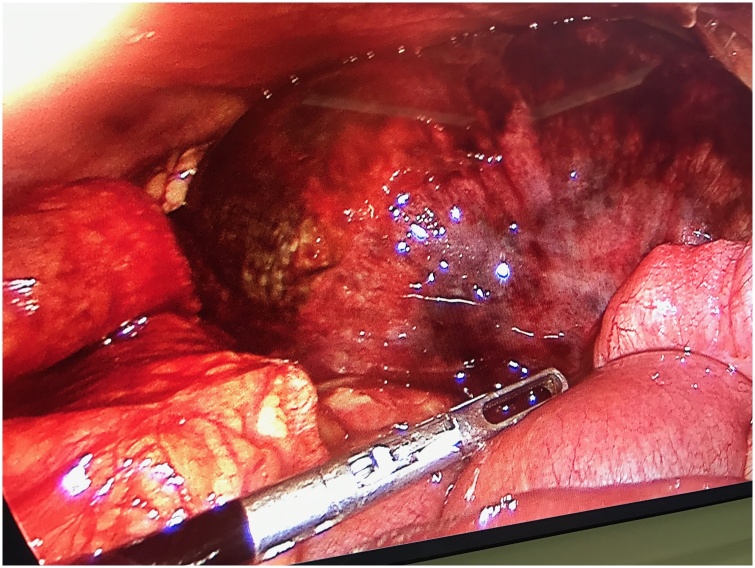
Ischemic segment of the terminal ileum, laparoscopic view.

**Fig. 3:**
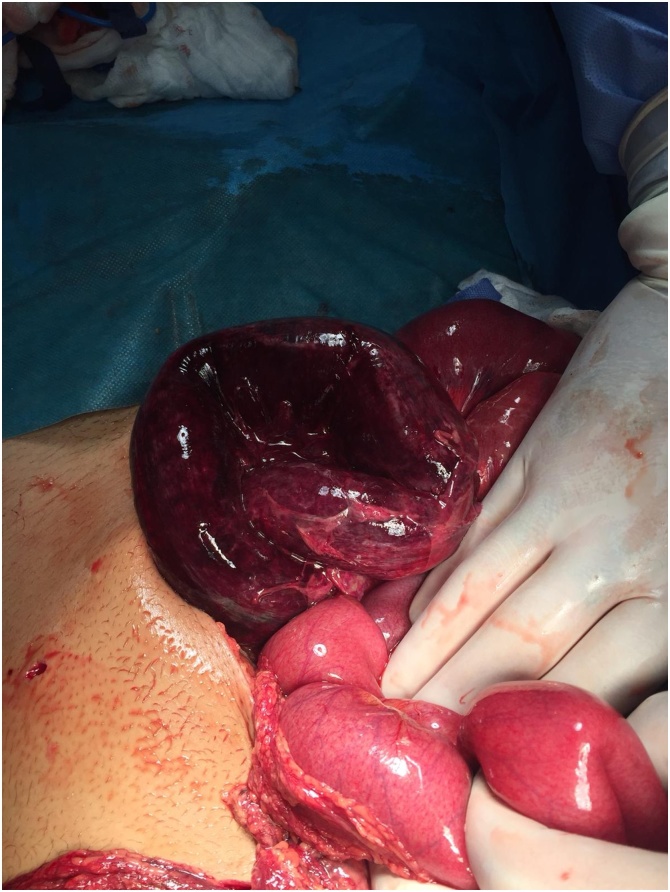
Ischemic segment of the terminal ileum, Laparotomy.

A 16-year-old male with unremarkable past medical or surgical history presented to the Emergency Department with progressively worsening abdominal pain and distention of 4 days’ duration. His vital signs were within normal limits. On physical examination, he had generalized abdominal tenderness and guarding of the abdomen. Laboratory findings were significant for an abnormally elevated white blood cell count of 24,900/mm3 but otherwise insignificant. A plain abdominal x-ray showed multiple air fluid levels. A computed tomographic scan showed a small amount of free fluid in the abdomen and dilated small bowel loops with evidence of ischemia (Fig. 1**A and B**). Based on this clinical presentation, the patient received an initial diagnosis of small intestinal obstruction and he was taken to the operating room. He was initially investigated with laparoscopy which revealed severe ischemic distal ileal segment. It was difficult to continue laparoscopically due to the severe small bowel dilatation (Fig. 2). We converted to a laparotomy and an ileocecectomy was performed (Fig. 3). During the procedure, approximately 20 cm of distal ileum was herniated through the superior ileocolic recess and were twisted along its mesentery. An ileocecectomy with a side to side primary anastomosis using a 75 GIA stapler was performed. The postoperative course was uneventful and the patient was discharged from the hospital on the 8th postoperative day.

## 3 Discussion

Internal hernia is the protrusion of visceral contents through a congenital or an acquired opening within the peritoneal cavity. It has an incidence of less than 1% and is significantly less common than external hernias [6]. According to the classification by Ghahremaniet al. [7], internal abdominal hernia can be of six main groups: paraduodenal, foramen of Winslow, transmesenteric, pericecal, intersigmoidal, and paravesical hernias.

Although internal hernias may have a congenital etiology, most of the reported cases have occurred during adulthood and presentation in childhood is uncommon [8]. The most common types of internal hernias that present in children are paraduodenal and transmesenteric hernias [9].

Pericecal hernias are responsible for 0.1–6.6% of internal hernias [3]. During embryological development, the anatomy of the cecal and pericecal peritoneum is not determined until the midgut has completed migration and the cecum is fixed in the right lower quadrant. Upon the resorption of peritoneal surfaces, four different pericecal recesses are formed by folds of the peritoneum - superior ileocecal recess, inferior ileocecal recess, retrocecal recess, and paracolicsulci [10]. These recesses may sometimes be absent or rarely large enough to admit several fingers and extend posterior to the cecum [3].

Upon literature review, three cases of pericecal hernia in the pediatric population were reported (Table 1).

**Table 1:** All reported cases of pericecal hernia in pediatrics (less than 18 years).

Age	Gender	Diagnosis	Intervention	Author/year
8 years	Male	Pericecal Hernia	Laparotomy	Rubin et al. [13]
8 weeks	Female	Pericecal Hernia	Laparotomy	Rivkind et al. [4]
8 weeks	Male	Pericecal Hernia	Laparotomy	Rivkind et al. [4]
Total cases	3			

Preoperative diagnosis of internal hernia is difficult because symptoms may range from intermittent and mild digestive complaints to symptoms of acute-onset intestinal obstruction. Internal hernias are silent if they are easily reducible, but the majority often cause epigastric discomfort, periumbilical pain, and recurrent episodes of intestinal obstruction [11]. Internal hernias are clinically apparent only when incarcerated internal hernias result from small bowel obstruction; therefore, a delay in diagnosis may lead to strangulation and an increased risk of serious complications [9].

Keeping in mind the risk of CT radiation, especially in children. CT scan is the key for internal hernia diagnosis and management as it provides information about the presence of bowel obstruction, strangulation, and ischemia. It aids in preoperative planning and when to urgently surgically intervene. CT appearance of a pericecal hernia is not established as there are few cases in the literature [10]. A CT scan usually reveals a cluster of bowel loops located posteriorly and laterally to the normal cecum, occasionally extending into the right paracolic gutter and pushing the cecum anteriorly and medially [6].

Treatment of an internal hernia with signs of bowel obstruction includes volume resuscitation, correction of electrolyte imbalances and surgery. Laparotomy has generally been the preferred approach for the presence of small bowel obstruction. Technical difficulties and potential complications such as having no work space from distended small bowel loops and the inability to visualize the transition point make laparotomy more preferred over laparoscopy. However, laparoscopic approach has shown safety and efficacy in select cases [12]. Benefits of laparoscopic surgery in small bowel obstruction are reduction in postoperative hospital stay, reduction in morbidity, reduction in adhesion formation and faster return to bowel function [12].

## 4 Conclusion

Pericecal hernia in a pediatric patient is rare but should be included in the differential diagnosis of small bowel obstruction. CT imaging is the imaging study of choice that aids in the diagnosis and management of pericecal hernia. High index of clinical suspicion and early intervention saves the patient from extensive bowel resection and other serious complications.

## Conflicts of interest

The authors declare that they have no conflict of interest regarding the publication of this case report

## Sources of funding

No funding was sought or secured in relation to this case report.

## Ethical approval

This is a case report, ethical approval not applicable.

## Consent

Written informed consent was obtained from the patient for publication of this case report and accompanying image. A copy of the written consent is available for review by the Editor-in-Chief of this journal on request.

## Author contribution

These authors contributed equally to this work. Loay M. Aljaberi wrote and reviewed the manuscript. AlaaEddin Salameh, Raed M. Mashalah, & Ayman AbuMaria reviewed the manuscipt.

## Registration of research studies

This is a case report, registration of research studies not applicable.

## Guarantor

Ayman Abu Maria, MD.

## Provenance and peer review

Not commissioned, externally peer-reviewed
